# Global Responses to the COVID-19 Pandemic

**DOI:** 10.3201/eid2813.221733

**Published:** 2022-12

**Authors:** Cynthia H. Cassell, Pratima L. Raghunathan, Olga Henao, Katina A. Pappas-DeLuca, Whitney L. Rémy, Emily Kainne Dokubo, Rebecca D. Merrill, Barbara J. Marston

**Affiliations:** Centers for Disease Control and Prevention, Atlanta, Georgia, USA

**Keywords:** COVID-19, coronavirus disease, SARS-CoV-2, severe acute respiratory syndrome coronavirus 2, viruses, respiratory infections, zoonoses, pandemic response, COVID-19 pandemic, global health, PEPFAR, surveillance systems, laboratory systems, information systems, workforce development, institutional development, public health capacity development, clinical and health services delivery and impact

Confronted with a novel coronavirus, countries worldwide were forced to rapidly adjust their public health systems, platforms, and tools to respond to COVID-19. The US Centers for Disease Control and Prevention (CDC) and its global partners adapted health systems and programs originally developed for other purposes, such as controlling the HIV/AIDS pandemic through the US President’s Emergency Plan for AIDS Relief (PEPFAR), Global Health Security Agenda implementation, influenza surveillance, and vaccine-preventable disease elimination and eradication. This special supplement of *Emerging Infectious Diseases* highlights responses to the early phases of the COVID-19 pandemic from >80 countries, spanning 6 continents and representing >130 organizations. This article summarizes global adaptations of core public health functions during COVID-19: surveillance, information, and laboratory systems; workforce, institutional, and public health capacity; and clinical and health services delivery.

## Surveillance, Information, and Laboratory Systems

CDC has provided longstanding support to strengthen surveillance, health information, and laboratory systems globally. Examples of such platforms used during the COVID-19 pandemic include the early warning and response surveillance system (*1*); respiratory (*2*), influenza (*3*), and acute febrile illness surveillance systems (*4*); global health security–supported information systems (e.g., District Health Information Software, version 2 [DHIS2]) (*5*); and PEPFAR-supported HIV and tuberculosis (TB) information systems (*6*,*7*). Respiratory disease surveillance guidance was developed for COVID-19 in 9 temporary camps for displaced persons along the Thailand–Myanmar border, showing that such systems can be effective during pandemics (*2*). Countries’ ministries of health (MOH), the World Health Organization (WHO), CDC, academic institutions, and nongovernmental organizations adapted international influenza surveillance systems for SARS-CoV-2 infections (*3*). CDC collaborated with MOH and partners to leverage existing acute febrile illness surveillance systems in 5 countries to collect and generate COVID-19 data needed for action (*4*). Kinkade et al. described 3 countries’ experience strengthening surveillance systems and reporting using DHIS2 for COVID-19 (*5*). Mirza et al. showed how health information systems for HIV and TB were modified for COVID-19 (*6*). PEPFAR-supported HIV and TB information management systems and diagnostic networks were adapted for SARS-CoV-2 testing in 16 low- to middle-income countries during the pandemic (*7*). Surveys provided key data on SARS-CoV-2 cases in Pakistan (*8*) and Malawi (*9*). Ohlsen et al. found international disparities in SARS-CoV-2 sequencing capacity and timeliness while viral genomic surveillance coverage increased globally (*10*). Smith-Sreen et al. compared 3 waves of the pandemic in 10 countries in southern Africa (*11*). Three neighboring countries in Africa used toolkits to analyze population movements and prioritize surveillance, cross-border collaboration, and communication strategies (*12*). Kenu et al. explained how geographic information systems were used for contact tracing to identify COVID-19 cases in Ghana (*13*). Chiou et al. developed a COVID-19 infodemic surveillance system to produce actionable insights to help address misinformation (*14*).

## Workforce, Institutional, and Public Health Capacity Development

CDC-supported Field Epidemiology Training Programs (FETPs) (*15*,*16*), Public Health Emergency Management (PHEM) Fellowships (*17*), and national public health institutes (NPHIs) (*18*) have contributed to leadership, disease detection and surveillance, and response and workforce capacity during the pandemic. Bell et al. described contributions to COVID-19 preparedness and response from 32 FETPs with 2,300 trainees and ≈7,400 graduates, representing >80 countries and 3 regions (*15*). Since 2013, CDC has offered the PHEM Fellowship to develop an international emergency response workforce; an assessment examined PHEM graduates’ roles during the pandemic (*17*). Zuber et al. reviewed the pivotal role NPHIs have played in pandemic response and identify gaps and priorities for further research (*18*).

Longstanding partnerships with MOH and other governmental bodies helped strengthen COVID-19 response capacity in Kenya (*19*), Nigeria (*20*), South Africa (*21*), and Cameroon (*22*). In Kenya, COVID-19 helped advance establishment of NPHIs and national and county-level emergency operations centers, workforce development and deployment, and training in surveillance, laboratory diagnostics, and infection prevention and control (IPC) (*19*). The Nigeria Presidential Task Force on COVID-19 worked with partners to develop a comprehensive National Pandemic Response Plan (*20*). In Cameroon, CDC’s global health programs were leveraged to respond to COVID-19, helping ensure continued delivery of HIV services and other health programs (*22*). Through PEPFAR, CDC used HIV Project Extension for Community Healthcare Outcomes programs, a model for virtual clinical mentorship, to address and assess healthcare workers’ response to COVID-19 (*23*). In 2021, the Public Health Center of Ukraine, Ukraine’s NPHI, engaged with faith communities to address public health measures during religious gatherings (*24*).

## Clinical and Health Services Delivery and Impact 

The pandemic also affected clinical and health services delivery. This supplement describes impacts on vaccine-preventable disease surveillance (*25*), expansion of COVID-19 vaccinations (*26*), and the effects of decreased hepatitis B immunization coverage (*27*). In the WHO Africa region, more than 200 Stop Transmission of Polio (STOP) Program consultants were surveyed to clarify how vaccine-preventable disease surveillance systems were disrupted during the pandemic (*25*). CDC’s COVID-19 International Vaccine Implementation and Evaluation program applied lessons learned from Ebola, influenza, and meningococcal serogroup A conjugate vaccine introductions for the delivery of COVID-19 vaccines (*26*). Experiences from past rubella vaccination programs (*28*), yellow fever and polio immunization campaigns for COVID-19 vaccine deployment and safety monitoring in Ghana (*29*), and the effectiveness of inactivated whole-virus COVID-19 vaccine among healthcare personnel in Peru (*30*) can also inform future responses. Zambia integrated COVID-19 vaccination at HIV treatment centers and combined activities planned for 2021 World AIDS Day to help increase vaccination outreach (*31*). Kimani et al. assessed IPC strategies and health facility readiness for responding to COVID-19 in Kenya, providing important data to guide IPC improvements (*32*). Gomes et al. described initiatives to strengthen IPC in healthcare facilities in 4 countries for the prevention of healthcare-associated transmission of SARS-CoV-2 (*33*).

COVID-19 affected other clinical services, including male circumcision for HIV prevention in sub-Saharan Africa (*34*) and care offered to survivors of sexual violence in Kenya (*35*). COVID-19 also caused clinical and socioeconomic impacts on agricultural workers in Guatemala (*36*). Protocols on community-based management of acute malnutrition in Uganda, Ethiopia, and Somalia needed modification to continue essential feeding services during the pandemic (*37*).

## Conclusion

International responses to COVID-19 demonstrated diverse adaptations, effects, and some improvements to public health systems and institutions; long-term global partnerships and collaborations across technical domains were central. The articles in this supplement issue contribute to ongoing efforts to stop outbreaks at their source and advance health equity to make the world safer, healthier, and more prepared for future public health emergencies.
